# Comparison of Nasopharyngeal MR, ^18^ F-FDG PET/CT, and ^18^ F-FDG PET/MR for Local Detection of Natural Killer/T-Cell Lymphoma, Nasal Type

**DOI:** 10.3389/fonc.2020.576409

**Published:** 2020-10-14

**Authors:** Rui Guo, Pengpeng Xu, Shu Cheng, Mu Lin, Huijuan Zhong, Weixia Li, Hengye Huang, Bingsheng Ouyang, Hongmei Yi, Jiayi Chen, Xiaozhu Lin, Kuangyu Shi, Weili Zhao, Biao Li

**Affiliations:** ^1^Department of Nuclear Medicine, Ruijin Hospital, School of Medicine, Shanghai Jiao Tong University, Shanghai, China; ^2^State Key Laboratory of Medical Genomics, Ruijin Hospital, School of Medicine, Shanghai Jiao Tong University, Shanghai, China; ^3^Siemens Healthcare, Beijing, China; ^4^Department of Radiology, Ruijin Hospital, School of Medicine, Shanghai Jiao Tong University, Shanghai, China; ^5^School of Public Health, Shanghai Jiao Tong University, Shanghai, China; ^6^Department of Pathology, Ruijin Hospital, School of Medicine, Shanghai Jiao Tong University, Shanghai, China; ^7^Department of Radiation, Rui Jin Hospital, School of Medicine, Shanghai Jiao Tong University, Shanghai, China; ^8^Department of Nuclear Medicine, University Hospital Bern, Bern, Switzerland; ^9^Faculty of Informatics, Technical University of Munich, Munich, Germany

**Keywords:** ^18^F-FDG, PET/MR, PET/CT, MR, extranodal NK/T-cell lymphoma, nasal-type

## Abstract

**Objectives:**

The present study aims to compare the diagnostic efficacy of MR, ^18^F-FDG PET/CT, and ^18^F-FDG PET/MR for the local detection of early-stage extranodal natural killer/T-cell lymphoma, nasal type (ENKTL).

**Patients and Methods:**

Thirty-six patients with histologically proven early-stage ENKTL were enrolled from a phase 2 study (Cohort A). Eight nasopharyngeal anatomical regions from each patient were imaged using ^18^F-FDG PET/CT and MR. A further nine patients were prospectively enrolled from a multicenter, phase 3 study; these patients underwent ^18^F-FDG PET/CT and PET/MR after a single ^18^F-FDG injection (Cohort B). Region-based sensitivity and specificity were calculated. The standardized uptake values (SUV) obtained from PET/CT and PET/MR were compared, and the relationship between the SUV and apparent diffusion coefficients (ADC) of PET/MR were analyzed.

**Results:**

In Cohort A, of the 288 anatomic regions, 86 demonstrated lymphoma involvement. All lesions were detected by ^18^F-FDG PET/CT, while only 70 were detected by MR. ^18^F-FDG PET/CT exhibited a higher sensitivity than MR (100% vs. 81.4%, χ^2^ = 17.641, *P* < 0.001) for local detection of malignancies. The specificity of ^18^F-FDG PET/CT and MR were 98.5 and 97.5%, respectively (χ^2^ = 0.510, *P* = 0.475). The accuracy of ^18^F-FDG PET/CT was 99.0% and the accuracy of MR was 92.7% (χ^2^ = 14.087, *P* < 0.001). In Cohort B, 72 anatomical regions were analyzed. PET/CT and PET/MR have a sensitivity of 100% and a specificity of 92.5%. The two methods were consistent (κ = 0.833, *P* < 0.001). There was a significant correlation between PET/MR SUVmax and PET/CT SUVmax (*r* = 0.711, *P* < 0.001), and SUVmean (*r* = 0.685, *P* < 0.001). No correlation was observed between the SUV and the ADC.

**Conclusion:**

In early-stage ENKTL, nasopharyngeal MR showed a lower sensitivity and a similar specificity when compared with ^18^F-FDG PET/CT. PET/MR showed similar performance compared with PET/CT.

## Introduction

Extranodal natural killer/T-cell lymphoma, nasal type (ENKTL) is a rare and distinct entity of extranodal non-Hodgkin lymphoma (NHL), which is more prevalent in Asia (1–3). ENKTL displays highly aggressive behavior with extensive local spread and poor prognosis. Since almost 75% of patients present early-stage I or II disease within the nasal cavity and adjacent sites (4), radiotherapy is routinely performed on these patients (5). Therefore, it is very important to define the extent of tumor invasion and observe regional structures in ENKTL to assess its complexity in the nasal cavity and surrounding areas (6–8).

Almost all cases of ENKTL are ^18^F-FDG avid (9, 10), and the efficacy of ^18^F-FDG PET/CT has been confirmed in the assessment of ENKTL (11–14). Magnetic resonance (MR) with diffusion-weighted imaging (DWI) has been reported to better distinguish the extent of tumor invasion in early-stage ENKTL owing to its ability to reveal fine anatomical details (15); however, the technique still warrants further validation. The combinational modality of PET/CT and MR is commonly recommended in patients with ENKTL for pretreatment evaluation and radiotherapy planning in National Comprehensive Cancer Network (NCCN) Guidelines (16). Whole-body ^18^F-FDG PET/MR was recently introduced into clinical imaging and offers a combination of metabolic information (provided by ^18^F-FDG PET) with a high soft tissue contrast anatomical resolution (provided by morphological MR). ^18^F-FDG PET/MR also provides an indirect assessment of cell density through use of DWI. PET/MR images demonstrate more discernible performance for mapping tumor invasion, particularly intracranial invasion, compared with PET/CT, and it is also effective in distinguishing retropharyngeal nodal metastases from adjacent nasopharyngeal tumors using a lower level of radiation (17). To our knowledge, the diagnostic efficacy of PET/MR in early-stage ENKTL has never been assessed.

In the present study, we investigated the efficacy of MR (including DWI), ^18^F-FDG PET/CT, and ^18^F-FDG PET/MR scans for detection of local lesions in patients with early-stage ENKTL and further explored if PET/MR is a viable alternative to the conventional PET/CT plus MR modality for pretreatment evaluations using a lower level of radiation.

## Materials and Methods

### Patients

From March 2014 to July 2017, 36 patients enrolled in a phase 2 study and an expansion cohort (NCT02825147, retrospectively analyzed) with newly diagnosed ENKTL in China underwent whole-body ^18^F-FDG PET/CT and nasopharyngeal MR (including DWI) within 14 days prior to the start of treatment (Cohort A). All patients had stage I or II disease. From May 2018 to November 2018, a further nine patients were prospectively enrolled from a multicenter, phase 3 study (NCT02631239) being conducted in China. These patients underwent whole-body ^18^F-FDG PET/CT followed by head and neck PET/MR (including DWI) before treatment with a single ^18^F-FDG injection (Cohort B). Patient diagnosis was defined using the World Health Organization classification (18). The study was approved by the Ethics Committee of Rui Jin Hospital, Shanghai Jiao Tong University, School of Medicine. Informed consent was obtained from patients in accordance with the Declaration of Helsinki.

### Examinations

^18^F-FDG PET/CT was performed using a Discovery VCT16 system (GE Healthcare, United States). The ^18^F-FDG tracer was manufactured automatically using the tracer synthesis system of the Tracerlab FXF-N (GE Healthcare), with a radiochemical purity >95%. Patients were required to fast for at least 6 h before imaging, and the serum glucose concentration was maintained at <7.0 mmol/L. A whole-body image was obtained 1 h after intravenous administration of 5–6 MBq of ^18^F-FDG per kilogram of body weight. CT was performed on the same scanner (120–180 mA and 140 kV). PET data was reconstructed using a three-dimensional attenuation-weighted ordered subset expectation maximization algorithm (Two iterations, 21 subsets, 256 × 256 matrix) and Gaussian smoothing kernel (full width at half maximum = 6 mm). MR imaging was performed using the 1.5-T system (Sigma, GE Healthcare, United States). All patients underwent an axial T1-weighted spin-echo sequence [repetition time (TR)/echo time (TE), 1709 ms/28 ms] and an axial T2-weighted fast spin-echo sequence (TR/TE, 4,800 ms/82 ms). DWI data were collected with tri-directional diffusion gradients (*b* values = 50, 800 s/mm^2^).

PET/MR was performed using an integrated PET/MR system (Biograph mMR; Siemens Healthineers, Erlangen, Germany). The PET/MR system was based on a 3.0-T MR system with a 16-channel radiofrequency head/neck coil. MR imaging was performed simultaneously with PET data acquisition with a total acquisition time of 15 min. The magnetic resonance imaging (MRI) protocol comprised the following sequences: high-resolution 3D magnetization-prepared rapid acquisition with gradient echo (3D-T1-MPRAGE) sequence (TR/TE, 4600 ms/66 ms); axial and coronal two-dimensional T2-weighted imaging with fat saturation (TR/TE, 740 ms/15 ms); and apparent diffusion coefficient (ADC) parametric maps based on single-shot DWI (*b* values = 50, 800 s/mm^2^). PET data was reconstructed as described above.

### Image Analysis

In Cohort A, MR images were analyzed by two radiologists with knowledge of histological diagnosis; both radiologists were blinded to the PET/CT results. PET/CT images were analyzed by two nuclear medicine physicians with knowledge of histological diagnosis; both physicians were blinded to the MR results. In Cohort B, PET/CT and PET/MR images were analyzed by two nuclear medicine physicians and two radiologists with knowledge of histological diagnosis; any differences in opinion were resolved by consensus. Eight nasopharyngeal anatomical regions in each patient, including nasal, nasopharynx, oropharynx/throat, sinus, bone, epidermal/soft tissue, eyelids/contents, and cervical lymph nodes, were imaged and assessed. A lesion with ^18^F-FDG uptake that was greater than normal radioactivity background or normal liver tissue and that was unrelated to the physiological site of tracer uptake or excretion was rated positive. MR images were assessed in terms of tumor mass enhancement, signal characteristics, location, local extension, bony destruction, soft tissue invasion, and regional lymph node involvement.

In Cohort B, the PET criteria for PET/MR were identical to the criteria for PET/CT. T1-weighted and T2-weighted MRI were used to anatomically locate the position of abnormal tracer accumulation in ^18^F-FDG PET. If DWI showed a high signal, and the corresponding ADC map showed a low value, the lesion was considered positive (19). Because false-positive findings in the lymph nodes have been previously reported with DWI, only lymph nodes with restricted diffusion and a long axis diameter of >1 cm were considered positive (20). A region of interest (ROI) analysis was performed based on ^18^F-FDG-PET images fused with images obtained from axial DWI. For each lesion, the maximum, mean, and peak standardized uptake value (SUVmax, SUVmean, and SUVpeak, respectively) of ^18^F-FDG PET/CT and ^18^F-FDG PET/MR, and the minimum and mean ADC (×10^–6^ mm^2^/s) of PET/MR were measured. ADC was measured on the section that showed the maximum transverse lesion diameter. Biopsy, additional imaging studies, and clinical follow-up were used as the gold standard to confirm lymphoma involvement in both cohorts.

### Statistical Analysis

To determine the diagnostic value of MR, ^18^F-FDG PET/CT, and ^18^F-FDG PET/MR, lesion-based sensitivity, specificity, and accuracy were calculated. Differences in sensitivity and specificity were determined using McNemar’s test. Spearman’s correlation coefficient (*r*) was used to assess the relationship between the SUV of ^18^F-FDG PET/MR and ^18^F-FDG PET/CT. The relationship of the SUV and the ADC of ^18^F-FDG PET/MR was also assessed. A *P*-value of <0.05 was considered statistically significant. All statistical tests were performed using SPSS Statistics 23.0 (SPSS Inc., Chicago, IL, United States).

## Results

### Patient Characteristics

In Cohort A, ^18^F-FDG PET/CT and nasopharyngeal MR were performed in 36 patients (Table 1). The median age was 49.5 years (age range, 13.0–72.0 years). In terms of clinical prognostic index, 18 (50.0%), 16 (44.4%), and 2 (5.6%) patients were defined as low-, intermediate-, and high-risk, respectively, using the prognostic index of natural killer lymphoma. The median interval between the beginning of PET/CT and MR was 3.5 days (range, 0–14 days). The median follow-up duration was 36.5 months (range, 7–57 months).

**TABLE 1:** Clinical characteristics of the patients in Cohort A (*n* = 36).

Characteristic	No. of patients	%
**Sex**		
Male	29	80.6
Female	7	19.4
**Age (y)**		
≤60	30	83.3
>60	6	16.7
**Ann Arbor stage**		
I	20	55.6
II	16	44.4
**Involvement of regional lymph nodes**		
Yes	7	19.4
No	29	80.6
**Prognostic index of natural killer lymphoma (PINK)**		
Low-risk (0)	18	50.0
Intermediate-risk (1)	16	44.4
High-risk (2–4)	2	5.6

In Cohort B, nine patients (six males and three females), with a median age of 44.0 years (range, 28.0–71.0 years) were analyzed (Table 2). The median follow-up duration was 18 months (range, 16–22 months). All patients had stage I or II disease within the nasal cavity and surrounding tissues. Cervical nodes were involved in four patients. The interval between the beginning of PET/CT and PET/MR was 42.9 ± 20.9 min (range, 25.0–79.0 min).

**TABLE 2:** Clinical characteristics of the patients in Cohort B (*n* = 9).

Characteristic	No. of patients	%
**Sex**		
Male	6	66.7
Female	3	33.3
**Age (y)**		
≤60	7	77.8
>60	2	22.2
**Ann Arbor stage**		
I	4	44.4
II	5	55.6
**Involvement of regional lymph nodes**		
Yes	4	44.4
No	5	55.6
**Prognostic index of natural killer lymphoma (PINK)**		
Low-risk (0)	7	77.8
Intermediate-risk (1)	2	22.2
High-risk (2–4)	0	0

### ^18^F-FDG PET/CT Scanning Versus MR

**FIGURE 1:**
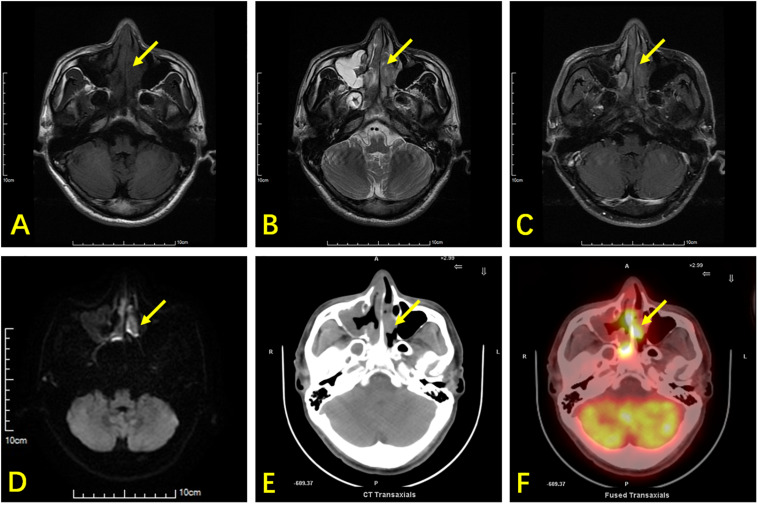
A 42-year-old man with ENKTL presented with nasal obstruction. **(A)** Axial section T1WI shows an isointense soft-tissue mass (arrow) in the nasal cavity and nasal septum. **(B)** Axial section T2WI shows the abnormal tissue as heterogeneously hyperintense. **(C)** Contrast-enhanced T1WI showing heterogeneous enhancement of tumor tissues. **(D)** DWI shows limited lesion diffusion. **(E)** Axial CT shows a soft tissue mass and nasal septum destruction. It was difficult to distinguish the soft tissue mass from inflammatory thickening of the mucosa in the right maxillary sinus using CT. **(F)** ^18^F-FDG PET/CT shows high glucose metabolism in the nasal cavity lesion, while low glucose metabolism is observed in the mucosa of the right maxillary sinus. The nasal cavity lesion was suspicious of tumor in both MRI and PET/CT, while the right maxillary sinus lesion was suspicious of inflammation.

In Cohort A, 288 sites in 36 patients were analyzed. There was at least one lesion with intense ^18^F-FDG uptake in all 36 patients, giving a patient-based detection rate of 100%, while MR detected at least one lesion in 31 out of 36 patients, giving a patient-based detection rate of 86.1%. The characteristics of ^18^F-FDG PET/CT and MR are illustrated in Figure 1. Of the 288 anatomical regions, 86 were positive for malignancy. ^18^F-FDG PET/CT detected all the malignant lesions, while MR detected 70 of the malignant lesions (sensitivity: 100% vs. 81.4%, χ^2^ = 17.641, *P* < 0.001). The specificities of ^18^F-FDG PET/CT and MR were 98.5 and 97.5% (χ^2^ = 0.510, *P* = 0.475), respectively. The accuracy of ^18^F-FDG PET/CT was 99.0%, and the accuracy of MR was 92.7% (χ^2^ = 14.087, *P* < 0.001). The two methods were consistent (κ = 0.927, *P* < 0.001). The detection efficiencies of nasopharyngeal ^18^F-FDG PET/CT and MR in patients with ENKTL in different regions are shown in Table 3.

**TABLE 3:** The efficacy of ^18^F-FDG PET/CT and ^18^F-FDG PET/MR in early-stage ENKTL in different nasopharyngeal anatomical regions.

	Method	No. of cases	Sensitivity (%)	Specificity (%)	Accuracy (%)
Nasal cavity	PET/CT	29	29/29 (100)	7/7 (100)	36/36 (100)
	MR	29	21/29 (72.4)	7/7 (100)	28/36 (77.8)
Nasopharyngeal	PET/CT	19	19/19 (100)	16/17 (94.1)	35/36 (97.2)
	MR	19	18/19 (94.7)	17/17 (100)	35/36 (97.2)
Oropharynx and throat	PET/CT	13	13/13 (100)	22/23 (95.7)	35/36 (97.2)
	MR	13	10/13 (76.9)	22/23 (95.7)	32/36 (88.9)
Sinus	PET/CT	8	8/8 (100)	28/28 (100)	36/36 (100)
	MR	8	7/8 (87.5)	28/28 (100)	35/36 (97.2)
Bone	PET/CT	2	2/2 (100)	34/34 (100)	36/36 (100)
	MR	2	2/2 (100)	34/34 (100)	36/36 (100)
Epidermal and soft tissue	PET/CT	5	5/5 (100)	31/31 (100)	36/36 (100)
	MR	5	4/5 (80)	31/31 (100)	35/36 (97.2)
Eyelids and contents	PET/CT	3	3/3 (100)	33/33 (100)	36/36 (100)
	MR	3	3/3 (100)	33/33 (100)	36/36 (100)
Cervical lymph nodes	PET/CT	7	7/7 (100)	28/29 (96.6)	35/36 (97.2)
	MR	7	5/7 (71.4)	25/29 (86.2)	30/36 (83.3)

*ENKTL, extranodal natural killer T-cell lymphoma, nasal type.*

**FIGURE 2:**
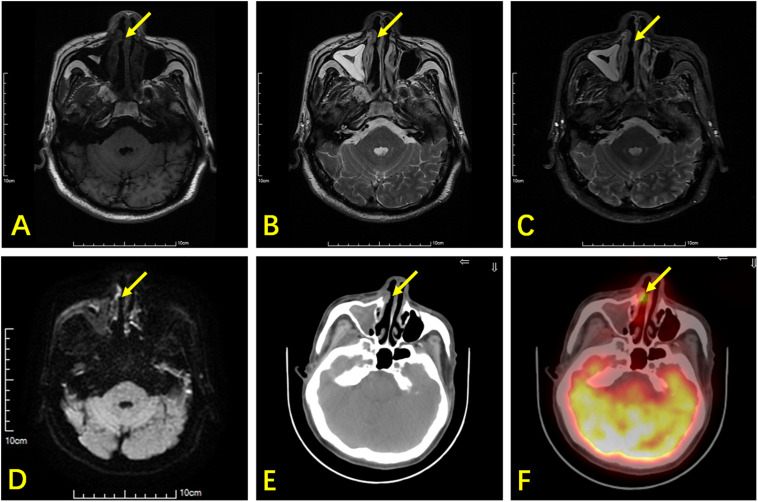
A 66-year-old man with ENKTL. T1WI **(A)** and T2WI **(B)** show bilateral thickening in the nasal mucosa with isointensity. **(C)** Contrast-enhanced T1WI show bilateral heterogeneous enhancement in the nasal mucosa. **(D)** DWI shows limited lesion diffusion of bilateral nasal mucosa. **(E)** Axial CT shows a small soft tissue mass (arrow) in the right nasal cavity. **(F)** ^18^F-FDG PET/CT shows high glucose metabolism in the small soft tissue mass, which was an ENKTL lesion, but this was non-specific in MR.

^18^F-FDG PET/CT revealed superior sensitivity over MR in 16 out of 86 lesions, including 5 lesions without any suspicious lesions in MR and 11 with lesion misinterpretations (Figure 2). The false negatives obtained using MR were mostly in the nasal cavity (8 out of 29, 27.6%) and oropharynx (3 out of 13, 23.1%). When sinusitis concurred, lymphoma was missed by MR.

There were three false-positive lesions when using ^18^F-FDG PET/CT. Two were caused by inflammation of cervical lymph nodes, which was confirmed at clinical follow up. The third was caused by physiological uptake in the oropharynx with an asymmetrical ^18^F-FDG concentration, which was later confirmed negative using a laryngoscope. All three lesions that were misrecognized by ^18^F-FDG PET/CT were negative on MR. However, five false-positive lesions were observed by MR. Four of these lesions were normal cervical lymph nodes with a high signal in DWI, while one was caused by nasal mucosal inflammation, which was confirmed as benign during clinical follow-up.

### Diagnostic Efficacy of ^18^F-FDG PET/CT and ^18^F-FDG PET/MR

**FIGURE 3:**
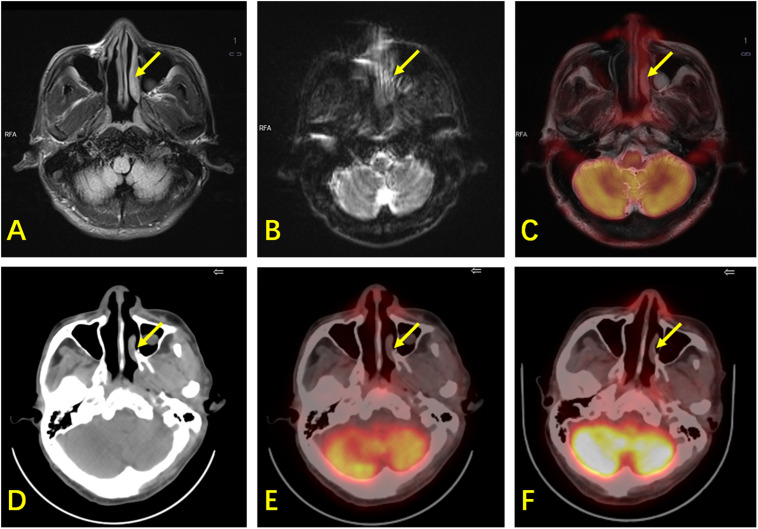
A 61-year-old man with ENKTL. **(A)** T2WI shows mild thickening of the nasopharynx and left nasal cavity mucosa (arrow) with hyperintensity. **(B)** DWI shows limited diffusion of the lesion. **(C)** PET/MR shows mild ^18^F-FDG uptake (SUVmax is 2.6, while the SUVmax of the reference liver is 2.3) and hyperintensity of T2WI of the lesion with suspected malignancy. **(D)** CT shows mild thickening of the nasopharynx and left nasal cavity mucosa. **(E)** ^18^F-FDG PET/CT shows mild ^18^F-FDG uptake in the nasopharynx (SUVmax is 1.9, while the SUVmax of the reference liver is 2.4), suspected as physiological. **(F)** There was still mild ^18^F-FDG uptake in the nasopharynx during follow-up. The patient did not show any sign of relapse after 42 months. This lesion was confirmed to be physiological in terms of its uptake, and was false-positive by PET/MR.

In Cohort B, the diagnostic efficacy of ^18^F-FDG PET/CT and PET/MR is shown in Table 4. Both PET/CT and PET/MR had high sensitivity (100% for both) and specificity (92.3% for both). There were three false-positive cases detected using PET/CT, which were in the nasopharynx, oropharynx, and cervical lymph nodes, respectively. These lesions still had mild ^18^F-FDG uptake during follow-up after chemotherapy, while other lymphoma lesions had disappeared, and all clinical lymphoma-related indicators improved, suggesting that the three sites were false positive. Since all cervical lymph nodes demonstrate a high signal with DWI, we combined lymph node morphology, ADC results, and FDG uptake to determine whether the lymph nodes were involved. However, there were still three false-positive lesions observed when using PET/MR, one in the cervical lymph nodes and the other two in the nasopharynx and the nasal cavity (Figure 3). A test for consistency in the diagnoses provided by PET/MR and PET/CT was conducted using McNemar’s test. The results suggest that the two methods were highly consistent (κ = 0.833, *P* < 0.001).

**TABLE 4:** Diagnostic efficacy of ^18^F-FDG PET/MR and ^18^F-FDG PET/CT in ENKTL.

		Pos	Neg	Total	Sen (95% CI), %	Spe (95% CI), %
PET/MR	Pos	32	3	35	100 (100)%	92.5 (84.3, 100)%
	Neg	0	37	37		
	Total	32	40	72		
PET/CT	Pos	32	3	35	100 (100)%	92.5 (84.3, 100)%
	Neg	0	37	37		
	Total	32	40	72		

*ENKTL, extranodal natural killer T-cell lymphoma, nasal type; Pos, number of positive examinations; Neg, number of negative examinations; Sen, sensitivity; Spe, specificity; CI, confidence interval.*

### The Correlation of SUVmax and SUVmean Between ^18^F-FDG PET/MR and ^18^F-FDG PET/CT

**FIGURE 4:**
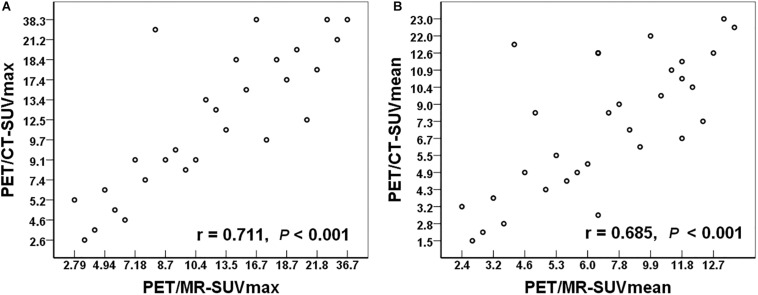
**(A)** The relationship of the SUVmax between PET/MR and PET/CT. **(B)** The relationship of the SUVmean between PET/MR and PET/CT. SUVmax, maximum standardized uptake value; SUVmean, mean standardized uptake value.

Among the 72 regions, a total of 32 regions were confirmed as malignant. We compared the SUV values (including SUVmax and SUVmean) in these lesions between PET/MR and PET/CT. There were no significant differences in SUVmax (*t* = −0.127, *P* = 0.900) or SUVmean (*t* = −1.202, *P* = 0.238) between PET/MR and PET/CT. Quantitative analysis and correlation analysis of the lesions are shown in Figure 4. There was a significant positive correlation in SUVmax between PET/MR (14.8 ± 8.6) and PET/CT (15.0 ± 9.8) (*r* = 0.711, *P* < 0.001). SUVmean also showed a positive correlation with PET/MR (8.0 ± 4.0) and PET/CT (8.9 ± 5.8) (*r* = 0.685, *P* < 0.001).

### The Correlation Between ADC and SUV in PET/MR

All of the 32 ^18^F-FDG-avid lesions exhibited restricted diffusion on DWI. The ADC value is an important quantitative indicator in DWI. In the 32 lesions, the mean ADCmin, ADCmean, SUVmax, SUVmean, and SUVpeak are illustrated in Table 5. We analyzed the correlation between the SUV and the ADC in ^18^F-FDG PET/MR. Pearson’s correlation analysis showed no significant correlation between the SUV and the ADC.

**TABLE 5:** The relationship between the ADC and the SUV in PET/MR.

SUV	Value	ADC	Value	*r*	*P*-value
SUVmax	14.8 ± 8.6	ADCmin	303.9 ± 226.8	−0.171	0.351
SUVmax	14.8 ± 8.6	ADCmean	722.3 ± 272.0	−0.083	0.650
SUVmean	8.0 ± 4.0	ADCmin	303.9 ± 226.8	0.03	0.869
SUVmean	8.0 ± 4.0	ADCmean	722.3 ± 272.0	−0.216	0.236
SUVpeak	11.3 ± 6.4	ADCmin	303.9 ± 226.8	−0.140	0.443
SUVpeak	11.3 ± 6.4	ADCmean	722.3 ± 272.0	−0.130	0.480

*ADC, apparent diffusion coefficients; SUV, standardized uptake value.*

## Discussion

Accurate staging plays an important role in the development of treatment plans for patients with ENKTL. In clinical practice, PET is necessary for whole-body detection of lymphoma involvement, while MR is required for local radiotherapy planning. To the best of our knowledge, the present study is the first to compare the efficacy of nasopharyngeal MR and nasopharyngeal ^18^F-FDG PET/CT in early-stage ENKTL in one cohort. This is also the first study to evaluate the role of PET/MR in this particular subtype of lymphoma.

In the present study, the nasal cavity and surrounding lesions were ^18^F-FDG-avid. PET exhibited high efficacy in cases with normal extranodal sites and normally sized lymph nodes, which is consistent with the results of previous studies (21–24). Our results indicate that ^18^F-FDG PET/CT is more efficacious than MR for lesion detection. Although MR may miss true lesions, including those in the nasal cavity or oropharynx that have no obvious morphological or signal changes; and may have false positivity (20), MR is still important for radiotherapy planning.

Integrated PET/MR is a new imaging modality with potential applications in oncology, such as lymphoma staging and treatment response evaluation. Giraudo et al. (25) compared ^18^F-FDG PET/CT and ^18^F-FDG PET/MR (including DWI) in the assessment of 34 patients with lymphoma. The sensitivity of PET/CT and PET/MR were 82.1 and 100%, respectively, while the specificity of both techniques was 100%. Four lesions were false negative in PET/CT. Our results show a sensitivity of 100% and a specificity 92.5% with both PET/CT and PET/MR. The relatively lower specificity of PET/MR in our study compared with Giraudo et al.’s study may be related to the characteristics of DWI. Since all cervical lymph nodes are high-signal in DWI (20), we considered multiple factors, including lymph node morphology, the ADC, and ^18^F-FDG uptake, to determine whether the lymph nodes are involved and to reduce incidence of false-positive results; however, three false-positive cases were still observed. The sensitivity of PET/CT in our study is higher than the sensitivity observed in Giraudo et al.’s study, which may be related to patient selection. The four false-negative results in their study were extranodal marginal zone B-cell lymphoma of mucosa-associated lymphoid tissue (MALT), which is an indolent lymphoma with poor ^18^F-FDG uptake, resulting in decreased sensitivity. DWI is considered superior in some indolent NHL subtypes (e.g., MALT lymphoma), which are frequently not ^18^F-FDG-avid. The patients enrolled in the present study were recruited from two prospective trials; all patients had stage I and II ENKTLs, which retained consistency and homogeneity in the study cohort.

The interval from the time of FDG administration to the time of imaging has an effect on the SUV (26). Hamburg et al. (27) found that the average time to reach 95% of the FDG plateau value was approximately 5 h in patients with lung carcinoma. The interval between the beginning of PET/CT and PET/MR was 42.9 ± 20.9 min in our study, so there may be a difference in the SUV. Other factors, such as MR-based attenuation correction and CT-based attenuation correction, may also have an effect on SUV (28). Therefore, there is no consensus on whether the SUV with PET/MR is higher or lower compared with PET/CT (28, 29). Drzezga et al. (29) reported that the SUVmean of lesions in PET/CT was greater than that of PET/MR, but the correlation between the two values was strong. Atkinson et al. (30) also showed a significant positive correlation between the SUVmax of PET/MR and the SUVmax of PET/CT. Many studies (28–30) are in agreement that the two methods demonstrate a good level of consistency. As a result, PET/MR may have a similar effect as PET/CT in terms of staging and efficacy evaluation. In the present study, we noticed no discrepancies in the SUV between PET/CT and PET/MR, which is consistent with previous studies.

The SUV reflects tumor glucose metabolism and tumor malignancy, while the ADC reflects the diffusion limitations of tumor cells and tissues. Both parameters are used to distinguish malignant lesions from non-malignant lesions. The relationship between the SUV and the ADC is interesting. The SUV and the ADC were negatively correlated in a previous study (31), while another study (32) did not identify a correlation between the ADC and the SUVmax in NHL in a comparative study. In the latter study (32), PET and MR were performed using different machines. Integrated PET/MR is useful to study the relationship between the SUV and the ADC and minimizes the error introduced by using different machines. Studies (30, 33) used integrated PET/MR to study different subtypes and stages of lymphoma, no correlation between the ADCmin and the SUVmax was observed. The present study used a consistent and homogeneous cohort of stage I and II ENKTL; no correlation was observed between ADCmin and SUVmax. The lack of correlation may indicate that their measures were reflective of two different physiologic qualities: metabolism (FDG) and cellular density (ADC). The relationship between glucose metabolism and cell density in ENKTL is undetermined and requires clarification with a large sample size. The lack of correlation in our study may be attributed to the generally recognized heterogeneity of tumor tissue (34). Finally, DWI and ADC values may have varied due to signal loss and motion artifacts in the neck region as a result of respiration and the arterial pulse, which is consistent with previous research (35). Despite the absence of a correlation between the SUV and ADC, we believe that DWI should be part of PET/MR protocols for lymphomas, since it has already demonstrated good diagnostic performance for this disease.

One limitation of the present study is that not all lesions (except of the primary site) that were classified as malignant underwent biopsy and pathological confirmation. Biopsies of every suggestive lesion are neither ethical nor recommended in routine clinical practice. Non-malignant lesions, such as focal inflammation, may be falsely regarded as positive. Another limitation of the present study is the small sample size used (20). However, to the best of our knowledge, this is the largest study to assess ENKTL independently, even in countries where this type of tumor is considered more prevalent. At the same time, due to the scarcity of PET/MR, research in this area is rare. Further large, multicenter, prospective studies are required to validate the diagnostic and staging efficacy of PET/MR for early-stage ENKTL.

## Conclusion

In conclusion, ^18^F-FDG PET/CT is more effective than MR for detection of local lesions in patients with early-stage ENKTL. PET/MR showed similar performance when compared with PET/CT.

## Data Availability Statement

The raw data supporting the conclusion of this article will be made available by the authors, without undue reservation.

## Ethics Statement

The studies involving human participants were reviewed and approved by the Ethics Committee of Rui Jin Hospital, Shanghai Jiao Tong University, School of Medicine. Written informed consent to participate in this study was provided by the participants’ legal guardian/next of kin.

## Author Contributions

RG performed the image analysis, collected and analyzed the data, and wrote the article. PX collected and analyzed the data, and wrote the article. SC and HZ collected the clinical data. ML designed PET/MR examinations. WL, XL, and KS performed the image analysis. HH was responsible for statistical review. BO and HY were responsible for pathology review. JC was responsible for the plan of patients’ radiotherapy. WZ and BL designed and supervised the study, and wrote the article. All authors contributed to the article and approved the submitted version.

## Conflict of Interest

ML was employed by Siemens Healthineers Ltd. The remaining authors declare that the research was conducted in the absence of any commercial or financial relationships that could be construed as a potential conflict of interest.
